# Type III Gustilo–Anderson open fracture does not justify routine prophylactic Gram-negative antibiotic coverage

**DOI:** 10.1038/s41598-023-34142-7

**Published:** 2023-05-01

**Authors:** Takashi Suzuki, Takahiro Inui, Miyoshi Sakai, Keisuke Ishii, Taketo Kurozumi, Yoshinobu Watanabe

**Affiliations:** 1 Trauma and Reconstruction Center, Teikyo University Hospital, Tokyo, Japan; 2 Department of Hygiene and Public Health, Teikyo University School of Medicine, Tokyo, Japan; 3 Trauma Center, Federation of National Public Service Personnel Mutual Aid Associations, Toranomon Hospital, Tokyo, Japan

**Keywords:** Medical research, Risk factors

## Abstract

Postoperative surgical site infection (SSI) is common in open long bone fractures, so early administration of prophylactic antibiotics is critical to prevent SSI. However, the necessity of initial broad-spectrum coverage for Gram-positive and -negative pathogens remains unclear. The purpose of this study was to clarify the effectiveness of prophylactic broad-spectrum antibiotics in a large, national-wide sample. We reviewed an open fracture database of prospectively collected data from 111 institutions managed by our society. A retrospective cohort study was designed to compare the rates of deep SSI between narrow- and broad-spectrum antibiotics, which were initiated within three hours after injury. A total of 1041 type III fractures were evaluated at three months after injury. Overall deep SSI rates did not differ significantly between the narrow-spectrum group (43/538, 8.0%) and broad-spectrum group (49/503, 9.8%) (*p* = 0.320). During propensity score-matched analysis, 425 pairs were analyzed. After matching, no significant difference in the SSI rate was seen between the narrow- and broad-spectrum groups, with 42 SSIs (9.9%) and 40 SSIs (9.4%), respectively (*p* = 0.816). The probability of deep SSI was not reduced by broad-spectrum antibiotics compared with narrow-spectrum antibiotics in type III open long bone fractures.

Early intravenous administration of prophylactic antibiotics in open fractures is currently the standard of care for prevention of surgical site infection (SSI)^1–7^. Antibiotics should be initiated as soon as possible after injury^8,9^, and specifically within 3 h at the latest^3,7,9,10^. In particular, Gustilo–Anderson type III open fractures have the highest risk of infection compared with the less severe type I and II fractures^11–13^. Gustilo et al. reported that 77% of cultures isolated from infected type III fractures showed Gram-negative pathogens, leading to the suggestion that a first-generation cephalosporin with addition of an aminoglycoside should be administered^12^. Over the last decade, there has been renewed interest in aminoglycoside-sparing regimens to reduce adverse effects, including acute kidney injury^3,4,7^. Most studies have reported no significant differences in infection rate compared with the use of aminoglycosides^4^. Accordingly, several guidelines have recommended Gram-negative coverage using broad-spectrum antibiotics with or without aminoglycosides in type III fractures^1–3^. However, there is little clinical evidence supporting the effectiveness of aminoglycosides or other broad-spectrum antibiotics^2,4^.

Most recently, several retrospective comparative studies have shown the prophylactic use of narrow-spectrum antibiotics for type III fractures without raising the infection rate^4,14–17^. Since those studies analyzed small cohorts, the lack of significant differences in infection rates was not necessarily remarkable. To clarify the controversies and provide real-world evidence, we conducted a retrospective, propensity score-matched cohort study of SSI in type III fractures using our society-managed open fracture registry. We hypothesized that early administration of broad-spectrum antibiotics in a large sample would reduce deep SSI, supporting previous guidelines.

## Methods

### Study design

**Figure 1:**
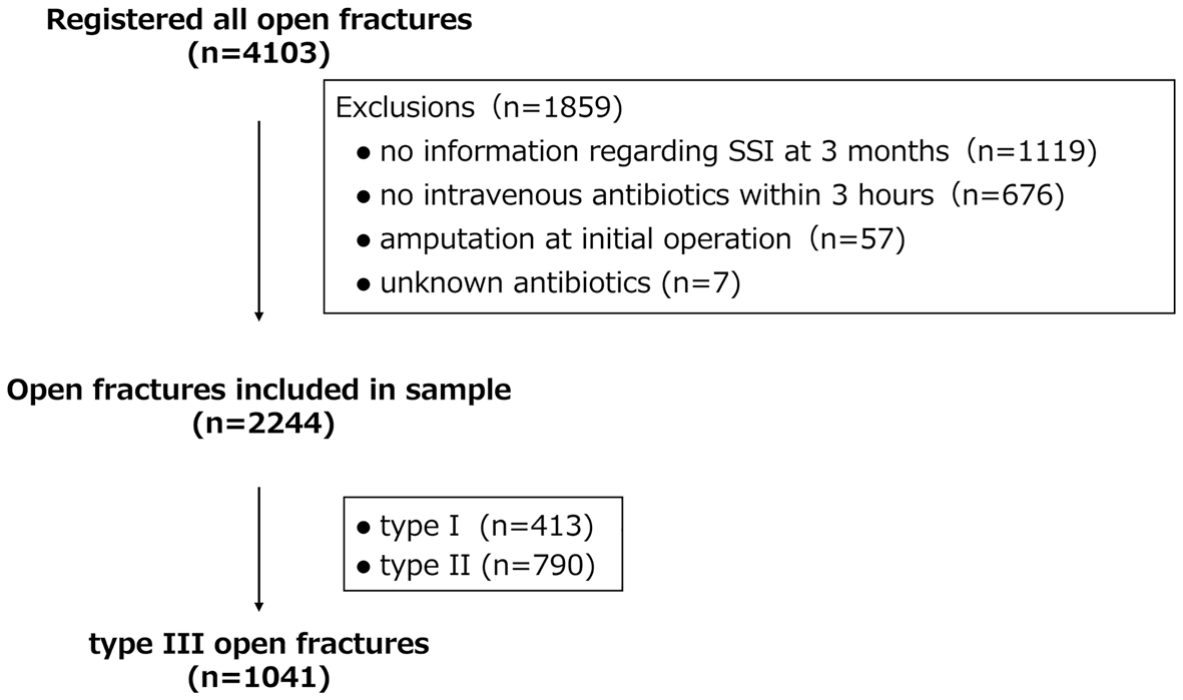
Flow diagram for inclusion and exclusion in the study. SSI: surgical site infection.

This analysis used data collected in the Database of Orthopaedic Trauma managed by the Japanese Society for Fracture Repair (DOTJ) registry conducted from February 2015 to June 2020. This registry obtained patient demographics, medical comorbidities, fracture classification, and operative variables as well as post-operative morbidities and functional outcomes collected in a prospective manner at the time of injury and at 3 months and 1 year after injury. The ad hoc registration committee of the society was responsible for the maintenance, validation, and cleansing of the data. The severity of open fractures was described according to both the Orthopaedic Trauma Association-Open Fracture Classification (OTA-OFC)^18^ and the Gustilo–Anderson classification^13^, as determined by the attending orthopaedic surgeons after surgical debridement. Enrollment took place in 111 institutions in Japan. The registry included patients who had sustained any open fractures in long bones (humerus, radius, ulna, femur, fibula, and tibia). Fractures of the hand, foot, and other axial fractures were excluded from registration. In total, 4103 open fractures in 3844 patients were registered to the DOTJ registry. The inclusion and exclusion criteria in this study are shown in Fig. 1. Fractures with no information regarding the presence or absence of SSI at 3 months after injury due to death of the patient or transfer to a rehabilitation facility were automatically excluded. Fractures with no intravenous antibiotic administration initiated within 3 h after injury were excluded. Fractures resulting in amputation in the initial operation were excluded to avoid the inclusion of amputation stump infections. Finally, fractures that were initially treated with unknown antibiotics at other hospitals were also excluded, leaving 2244 open fractures available for follow-up. These comprised 413 type I, 790 type II, and 1041 type III fractures. Our final cohort consisted of the 1041 type III fractures.

The definition of SSI was based on the Centers for Disease Control (CDC) and Prevention / National Nosocomial Infections Surveillance system^19^. This definition includes deep SSI with an onset within 90 days postoperatively.

### Prophylactic antibiotics

**Figure 2:**
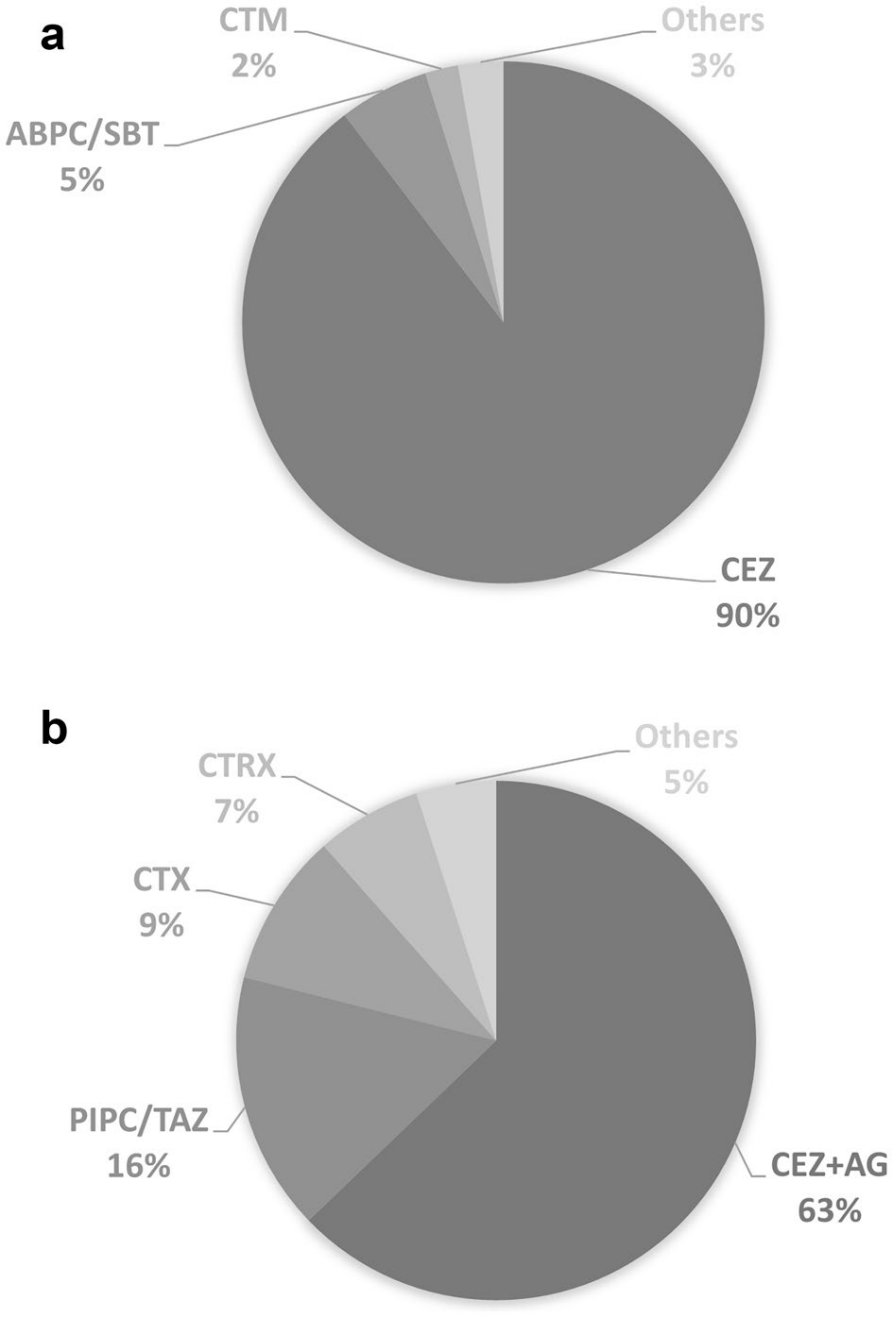
Distribution of commonly prescribed antibiotic combinations for open fractures in the narrow-spectrum group (**a**) and broad-spectrum group (**b**). CEZ: cefazolin; ABPC/SBT: ampicillin/sulbactam; CTM: cefotiam; AG: aminoglycoside; PIPC/TAZ: piperacillin/tazobactam; CTX: cefotaxime; CTRX: ceftriaxone.

Of the 1041 type III fractures, 538 were classified as the narrow-spectrum group and the remaining 503 were classified as the broad-spectrum group. Here, narrow spectrum means antibiotics that mainly target Gram-positive cocci, involving first- and second-generation cephalosporins, clindamycin as well as penicillin without covering *Pseudomonas*. Broad spectrum means antibiotics covering combined Gram-positive cocci and Gram-negative rods. Figure 2 shows the distribution of prophylactic antibiotics initiated within 3 h after injury. In the narrow-spectrum group, the most commonly prescribed antibiotic was cefazolin only (482 fractures; 89.6%), followed by ampicillin/sulbactam (30 fractures; 5.6%). Broad-spectrum antibiotics included aminoglycosides, third- and fourth-generation cephalosporins, penicillin with activity against *P. aeruginosa*, fluoroquinolones, monobactams, and carbapenems. The combined use of narrow- and broad-spectrum antibiotics was categorized into the broad-spectrum group. In the broad-spectrum group, 62.8% received cefazolin plus aminoglycosides. The most common aminoglycoside accompanying cefazolin was gentamicin (131 fractures; 26.0%), followed by amikacin (110 fractures; 21.9%). Detailed antibiotic regimens for each group are provided in the Supplementary Table S1. Ten different combinations of antibiotics in the narrow-spectrum group and 42 different combinations in the broad-spectrum group were prescribed before initial surgical debridement, similar to the situation reported by Lin et al.^6^.

### Statistical analysis

Using a difference in SSI rates of 4% based on previous studies^20^, an alpha level of 0.05, and power of 0.80, the total projected sample size needed was approximately 626 (313 per arm).

Univariate analyses were performed using the chi-square test for categorical variables and Student’s t test for continuous variables to assess differences in demographic characteristics between patients in the narrow- and broad-spectrum groups.

We performed one-to-one propensity score-matched analysis for the administration of broad-spectrum antibiotics. We selected patient demographic characteristics and other potential confounding variables derived from previous studies^8,21–23^. Those factors included age, sex, body mass index (BMI), American Society of Anesthesiologists (ASA) class, smoking, diabetes mellitus, time to completion of debridement, open fracture location (i.e., upper vs. lower extremity), Gustilo–Anderson classification, and summative score of the OTA-OFC. The covariate balance resulting from propensity score matching was checked using standardized differences. The maximum standardized mean difference between propensity probabilities for matching was set at 0.1. Balance was found to be adequate without restricting which patients were matched by enforcing a matching caliper width of 0.005.

All statistical analyses were performed using IBM SPSS Statistics software (version 28.0; IBM, Chicago, Illinois/USA). The level of significance was set at *p* < 0.05.

### Ethical approval

All procedures performed were in accordance with the ethical standards of the institutional and/or national research committee and with the 1964 Helsinki declaration and its later amendments or comparable ethical standards. This study was approved by the Institutional Review Board (Ethical committee approval No. 18-010).

### Informed consent

The need to obtain informed consent from the involved patients was waived by the institutional review board. (Ethical Review Board of Teikyo University).

## Results

**Figure 3:**
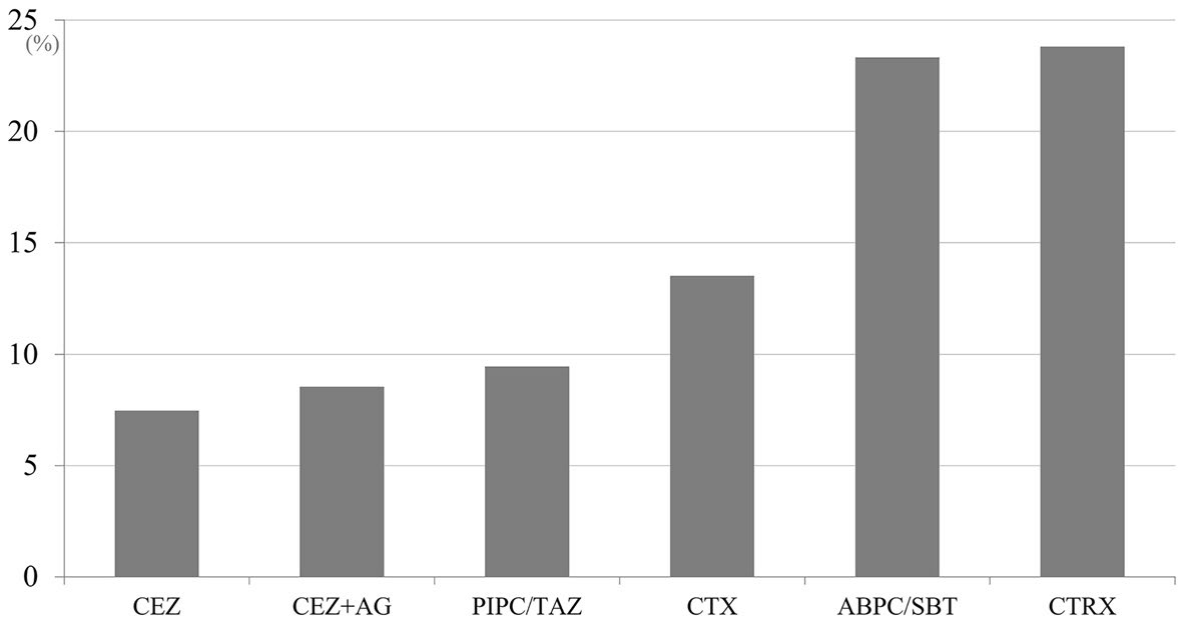
Crude surgical site infection rates for major antibiotic regimens. CEZ: cefazolin; AG: aminoglycoside; PIPC/TAZ: piperacillin/tazobactam; CTX: cefotaxime; ABPC/SBT: ampicillin/sulbactam; CTRX: ceftriaxone.

The unmatched cohort showed 92 open fractures with deep SSI. The crude SSI rates for major antibiotic regimens are provided in Fig. 3. Overall rates of deep SSI were 8.0% (43 of 538) in the narrow-spectrum group and 9.7% (49 of 503) in the broad-spectrum group. Chi-square analysis found no significant difference in overall SSI rate between groups (*p* = 0.320). There also showed no significant difference in SSI rate between cefazolin and cefazolin plus an aminoglycoside (Supplementary Table S2). Table 1 shows the fracture characteristics of the cohort of patients prior to matching. As expected, patients administered broad-spectrum antibiotics showed more severe prognostic factors, including fractures with type IIIB and IIIC classification as well as higher OTA-OFC scores.

**Table 1:** Characteristics of fractures administered with narrow and broad spectrum antibiotics (unmatched).

	Narrow (N = 538)	Broad (N = 503)	Standardized mean difference	*p* Value
Age† (yr)	51.1 ± 19.3	50.3 ± 19.1	0.041	0.508
Male sex	385 (71.6%)	382 (75.9%)	0.100	0.121
BMI† (kg/m^2^)	22.97 ± 4.0	22.79 ± 3.9	0.047	0.452
ASA ≥ 3	74 (13.8%)	86 (17.1%)	0.093	0.144
Diabetes mellitus	41 (7.6%)	35 (7.0%)	0.025	0.721
Smoking	178 (33.1%)	187 (37.2%)	0.086	0.173
Gustilo type			0.247	< 0.001
IIIA	363 (67.5%)	280 (55.7%)		
IIIB	136 (25.3%)	168 (33.4%)		
IIIC	39 (7.2%)	55 (10.9%)		
OTA-OFC summative score†	7.8 ± 2.2	8.4 ± 2.4	0.295	< 0.001
Fracture location			0.067	0.285
Upper extremity	143 (26.6%)	119 (23.7%)		
Lower extremity	395 (73.4%)	384 (76.3%)		
Completion of debridement > 12 h	49 (9.1%)	36 (7.2%)	0.071	0.26

BMI: body mass index, ASA: American Society of Anesthesiologists, OTA-OFC: Orthopedic Trauma-Open Fracture Classification.

^†^The values are given as the mean and the standard deviation.

After propensity score matching, 425 matched pairs of fractures were available for comparison. Both groups showed similar characteristics among all covariates (Table 2). The rate of deep SSI was 9.9% (42/425) in the narrow-spectrum group, compared with 9.4% (40/425) in the broad-spectrum group (*p* = 0.816).

**Table 2:** Characteristics of fractures administered with narrow and broad spectrum antibiotics (matched).

	Narrow (N = 425)	Broad (N = 425)	Standardized mean difference	*p* Value
Age† (yr)	50.0 ± 19.0	50.62 ± 19.4	0.030	0.657
Male sex	331 (77.9%)	324 (76.2%)	0.039	0.625
BMI† (kg/m^2^)	22.7 ± 3.8	22.88 ± 3.9	0.056	0.412
ASA ≥ 3	66 (15.6%)	68 (16.1%)	0.013	0.925
Diabetes mellitus	27 (6.4%)	30 (7.1%)	0.028	0.784
Smoking	157 (36.9%)	156 (36.7%)	0.005	1.000
Gustilo type			0.094	0.405
IIIA	284 (66.8%)	265 (62.4%)		
IIIB	114 (26.8%)	129 (30.4%)		
IIIC	27 (6.4%)	31 (7.3%)		
OTA-OFC summative score†	8.1 ± 2.2	8.0 ± 2.1	0.031	0.654
Fracture location			0.070	0.345
Upper extremity	115 (27.1%)	102 (24.0%)		
Lower extremity	310 (72.9%)	323 (76.0%)		
Completion of debridement > 12 h	28 (6.6%)	32 (7.5%)	0.037	0.688

BMI: body mass index, ASA: American Society of Anesthesiologists, OTA-OFC: Orthopedic Trauma-Open Fracture Classification.

^†^The values are given as the mean and the standard deviation.

**Figure 4:**
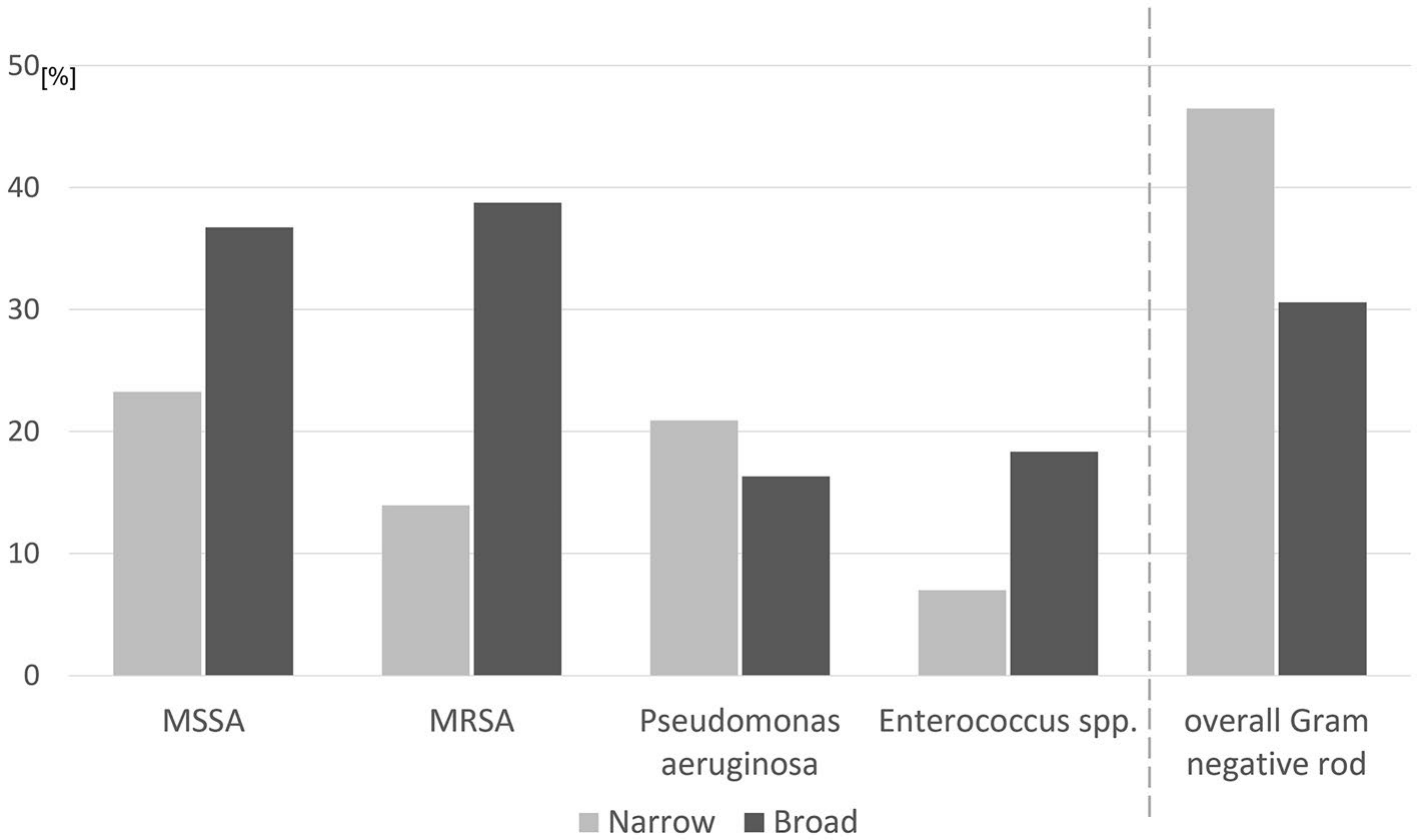
Main culture results and prevalence of overall Gram-negative rod pathogens for surgical site infections in unmatched cohorts. MSSA: methicillin-susceptible *Staphylococcus aureus*; MRSA: methicillin-resistant *Staphylococcus aureus*.

For culture data, the unmatched cohort showed 12/43 (27.9%) polymicrobial infections in the narrow-spectrum group and 18/49 (36.7%) polymicrobial infections in the broad-spectrum group, with 57 and 79 pathogens in total, respectively. The most frequent pathogens in each group before matching are shown in Fig. 4. *Staphylococcus* species were identified in 41.9% (18/43) of the narrow-spectrum group and 83.7% (41/49) of the broad-spectrum group (*p* < 0.001). Rates of occurrence of overall Gram-negative rod pathogens were 46.5% (20/43) in the narrow-spectrum group and 30.6% (15/49) in the broad-spectrum group (*p* = 0.117). After propensity score matching, *Staphylococcus* species were identified in 42.9% (18/42) of the narrow-spectrum group and 80.0% (32/40) of the broad-spectrum group (*p* < 0.001). Rates of overall Gram-negative rod pathogens were 45.2% (19/42) in the narrow-spectrum group and 32.5% (13/40) in the broad-spectrum group (*p* = 0.237).

## Discussion

The necessity of coverage against Gram-negative pathogens has remained controversial in the initial treatment of type III open fractures. Although previous guidelines and articles recommend the use of broad-spectrum antibiotics such as aminoglycosides, third- or fourth-generation cephalosporins or broad-spectrum penicillin with activity against *P.* *aeruginosa*^1–3,5,12,13,24–26^, surprisingly no studies in the literature appeared to have directly compared Gram-positive coverage to combined Gram-positive and Gram-negative coverage in terms of deep SSI rates at the time of the International Consensus Meeting on Musculoskeletal Infection held in 2018^2^. The present study using propensity score-matched analysis demonstrated no difference in deep SSI rates between the use of narrow- and broad-spectrum antibiotics when comparing 425 fractures in each group.

Several authors have reported an increased risk of infection with Gram-negative pathogens following type III fractures. Gustilo et al. reported that Gram-negative pathogens comprised three-quarters of cultured bacteria in type III fractures and advocated the addition of aminoglycosides as prophylactic antibiotics^12^. Dellinger et al. used second-generation cephalosporins for prophylaxis and reported that 48% of infections were caused by Gram-negative pathogens^27^. Vasenius et al. reported the efficacy of Gram-positive coverage with agents such as clindamycin and cloxacillin^28^. They revealed that those antibiotics were effective for type I and II fractures, but SSI rates were high for type III fractures with both clindamycin (29.0%) and cloxacillin (51.8%). The recommendations of the guidelines rely primarily on those studies^1–3^, which simply showed increased rates of Gram-negative infection in type III fractures. Although we understand the rationale behind efforts to reduce SSIs caused by Gram-negative pathogens, the recommendation for prophylactic use of broad-spectrum antibiotics has not been supported by objective evidence.

It is not yet completely understood why Gram-negative pathogens take over from Gram-positive pathogens in type III fracture infection. Previous studies have shown that causative organisms differed from those found in the initial wound cultures^4,21,29–33^. It seems apparent that results from bacterial cultures taken intraoperatively after debridement are not predictive of pathogenic bacteria causing postoperative infection^5^. Traditionally, the standard strategy for type III fractures has been to avoid primary wound closure^10–12^. The wounds in type III fractures have thus been managed by delayed wound closure until several days post-injury to allow drainage of any slowly accumulating infectious materials^34,35^. Naturally, the profiles of the causative organisms differ, with a higher proportion of Gram-negative species being associated with wounds closed in a delayed fashion^7^. Jenkinson et al. clearly demonstrated that Gram-negative pathogens increased more in a delayed wound closure group compared with a primary closure group, even for open fractures with the same severity^35^. Konda et al. reported that hospitalization for longer than 10 days and a need for soft tissue coverage of an open fracture site represented independent risk factors for Gram-negative infection^36^. Infection in type III fractures is related to the extent of soft tissue damage and time to wound coverage, but not to the choice of prophylactic antibiotics^8,21^. Open fracture infections are predominantly nosocomial and therefore are mainly caused by Gram-negative pathogens^33^.

Antibiotic coverage for nosocomial and multidrug-resistant organisms is an ongoing area of clinical research^3–7,20,26^. The duration of antibiotic administration is also under discussion^4,6,7^, although a recent systematic review of randomized controlled trials and several other studies found no difference between durations of 1 versus 3–5 days, even in type III fractures^2,3,5,21^. Some authors have suggested that the use of broader antibiotic coverage for a prolonged duration cannot guarantee the absence of subsequent infection because pathogens will be selected for by the administered antibiotics^7,21^. We think that the antibiotics chosen for prophylaxis were not sufficiently effective in protecting damaged soft tissues from subsequently developing nosocomial infection. Even if administration of broad-spectrum antibiotics might reduce some types of Gram-negative infection, growth of other pathogens cannot be completely avoided, which could result in little change to the overall infection rate.

Most recently, several investigators have reported that narrow-spectrum antibiotics were sufficient for type III fractures, with no increased risk of deep infection. Bankhead-Kendall et al. reported patients with type III fractures treated using cephalosporin (n = 65) versus cephalosporin plus an aminoglycoside (n = 61)^14^. They noted that the SSI rate did not differ between groups. Takahara et al. compared ampicillin/sulbactam (n = 34) with cefazolin plus aminoglycosides (n = 56) in type IIIA fractures^15^. They demonstrated the non-inferiority of ampicillin/sulbactam with respect to deep SSI rate. Depcinski et al. reported on a single-center review of patients with type III fractures who were treated with cefazolin (n = 53) versus cefazolin plus an aminoglycoside (n = 15)^16^. The rate of infection was higher in the cefazolin plus aminoglycoside group, in which causative organisms were more commonly multi-drug resistant pathogens. Patanwala et al. performed a multicenter study of patients with type III fractures^17^, finding no difference in 30-day infection rates between the cefazolin (n = 39) and cefazolin plus aminoglycosides (n = 95) group. Whereas those studies were based on small sample sizes, some recent reviews have described cefazolin monotherapy as being potentially as effective as broad-spectrum antibiotics^4,6^. Our findings in the present study are in line with those studies.

A number of limitations need to be considered when interpreting the results of our study. One weakness was the poor interobserver reliability of the Gustilo–Anderson classification system^22^. The registered classification was determined after surgical debridement. Although all participating institutions had sufficient clinical experience to perform adequate evaluation, the OTA-OFC score was selected as a covariate of propensity score matching to control between-hospital differences, thus helping to improve the characterization of severity for open fractures^22^. Second, the loss to follow-up could have compromised the validity of the study. Once enrolled in the DOTJ registry, each institution prospectively registered treatment details and outcomes. Due to the inherent nature of prospectively maintained trauma registries, patients lost to follow-up due to patient death or transfer inevitably increased with longer duration of follow-up. Third, we did not consider independent variables including surgical procedures such as performance of external fixation, timing of flap coverage, or definitive internal fixation, since the analyses of these showed inadequate statistical power. Judging from the distribution of various strategies and complex treatment courses for each open fracture, categorizing these factors into specific subgroups was difficult. Unified, pre-defined treatment protocols are needed to allow specialized subgroup analyses. Finally, the data are from real-world observations, and unknown or non-measurable confounders could not be assessed. Further, factors contributing to the immune status of patients, such as age, lifestyle factors, and comorbidities, may influence the occurrence of surgical site infection. In particular, multiple combinations of different antibiotics may have affected the analyses^6^. The use of therapeutic antibiotics may have been driven by concomitant severe injuries. Antibiotic choice may be affected by the underlying disease. Additional prospective studies are needed to truly determine what risk factors advocate the use of prophylactic Gram-negative antibiotic coverage in type III fractures.

Despite these limitations, the present study detected no reduction in deep SSI rate with broad-spectrum antibiotics, compared to narrow-spectrum antibiotics on the basis of a much larger sample size compared with the prior literature. Since all open fractures were included in the registry regardless of the level of the trauma center, the present study reflected the real-world prevalence of open long bone fractures. Contrary to previous guidelines, a Gustilo–Anderson type III classification alone did not justify routine use of prophylactic Gram-negative antibiotic coverage. We need to take into consideration a wide variety of important factors such as cost-effectiveness, selection of antimicrobial resistance, and drug adverse effects in addition to risks of SSI^16,37^.

## Conclusions

Our propensity score-matched analysis demonstrated no prophylactic benefit from Gram-negative coverage with broad-spectrum antibiotics in Gustilo–Anderson type III open fractures in terms of the development of deep SSI within 3 months. Even if broad-spectrum antibiotic regimens were employed for prophylaxis, the prevalence of Gram-negative rod pathogens causing SSI was not significantly reduced among cases with type III fractures overall.

## Supplementary Information

Supplementary Information 1.

Supplementary Information 2.

## Data Availability

The corresponding author can be contacted and anonymized data will be shared on request from any qualified investigator upon reasonable request.
